# Antioxidants in Cardiovascular Medicine: Emerging Trends and Future Perspectives

**DOI:** 10.3390/antiox15081001

**Published:** 2026-08-12

**Authors:** Claudia Fiorillo, Matteo Becatti

**Affiliations:** Department of Experimental and Clinical Biomedical Sciences “Mario Serio”, University of Firenze, 50134 Firenze, Italy; claudia.fiorillo@unifi.it

## Introduction

Cardiovascular diseases remain among the principal causes of morbidity, disability, and mortality worldwide [1,2]. Despite remarkable advances in lipid-lowering therapy, antithrombotic treatment, neurohormonal modulation, and the management of cardiometabolic risk factors, a substantial residual cardiovascular risk persists [3,4,5,6,7]. This residual burden reflects the biological complexity of cardiovascular disease, which cannot be explained by a single pathway or confined to a single organ [8,9]. Rather, cardiovascular disorders arise from the interaction of dyslipidemia, chronic inflammation, endothelial dysfunction, mitochondrial impairment, metabolic dysregulation, and thrombosis [10,11,12]. Redox imbalance is deeply embedded within each of these processes and contributes to vascular inflammation, thrombotic activation, and tissue injury across several human diseases [13,14,15,16,17,18], providing a unifying mechanistic framework through which apparently distinct cardiovascular phenotypes can be interpreted.

Reactive oxygen species (ROS) and reactive nitrogen species (RNS) are frequently portrayed as exclusively detrimental molecules. However, this view does not adequately reflect their physiological roles [19]. At controlled concentrations, reactive species act as signaling mediators that regulate vascular tone, mitochondrial adaptation, cellular proliferation, immune responses, angiogenesis, and tissue repair, highlighting the dual physiological and pathological role of redox signaling that has also emerged in several inflammatory and noncardiovascular disease models [13,20,21]. For example, nitric oxide is essential for endothelial homeostasis, platelet inhibition, and the regulation of vascular smooth muscle tone [22,23]. Similarly, transient ROS production participates in the cellular responses to metabolic and mechanical stress [24].

The pathological consequences of reactive species emerge when their generation exceeds the capacity of endogenous antioxidant systems or when their spatial and temporal regulation is disrupted. Under these conditions, redox signaling is replaced by oxidative or nitrosative damage [25]. Lipids, nucleic acids, carbohydrates, and proteins become targets of irreversible modification, whereas redox-sensitive signaling pathways sustain inflammation, endothelial activation, mitochondrial dysfunction, and maladaptive tissue remodeling. Accordingly, accurately assessing oxidative status has become increasingly important in clinical research on different human diseases [26,27,28]. The biological impact is determined not simply by the total amount of reactive species produced but also by their chemical identity, cellular source, localization, duration of exposure, and molecular targets.

This complexity helps explain why conventional antioxidant supplementation has often produced inconsistent results in cardiovascular clinical trials. The indiscriminate removal of reactive species may fail to reach the relevant cellular compartment, may not suppress the enzymatic source of pathological oxidants, and may interfere with physiological redox signaling [29,30,31,32]. Consequently, contemporary antioxidant medicine is moving away from the concept of nonspecific free-radical scavenging and toward a more sophisticated strategy based on the modulation of endogenous defense pathways, mitochondrial function, inflammatory signaling, nitric oxide bioavailability, and the enzymatic sources of ROS and RNS. This paradigm shift reflects the growing recognition that redox biology should be investigated through targeted mechanisms and validated biomarkers rather than through indiscriminate antioxidant supplementation [13,26].

The contributions collected in this Special Issue, “Antioxidants in Cardiovascular Medicine: Emerging Trends and Future Perspectives”, reflect this transition. This collection comprises three reviews and three original research articles spanning molecular mechanisms, thrombosis, atherosclerosis, heart failure, circulating biomarkers, nutraceutical interventions, gut-microbiota-derived metabolites, and life-course cardiovascular prevention. Together, these studies illustrate how redox biology can connect mechanistic discovery with biomarker development and therapeutic innovation.

This collection begins with a comprehensive review of the relationship between RNS and fibrinogen nitration (Contribution 1). This subject provides a compelling example of how nitro-oxidative stress can translate into clinically relevant alterations in hemostasis. Fibrinogen is not merely a soluble precursor of fibrin but a highly organized plasma glycoprotein whose structure governs clot formation, fibrin architecture, mechanical stability, platelet interactions, and fibrinolysis [33]. Its modification by RNS may therefore influence the initiation and resolution of thrombotic events. The authors focus particularly on peroxynitrite, generated through the rapid reaction between nitric oxide and superoxide [34]. Peroxynitrite and related nitrating species modify the tyrosine residues in the functional regions of the Aα, Bβ, and γ fibrinogen chains. Such modifications can produce conformational changes, dityrosine crosslinking, and the formation of high-molecular-weight aggregates. The resulting fibrin networks may display abnormal polymerization, increased structural heterogeneity, altered porosity, and reduced mechanical strength. The effects on fibrinolysis appear to be dependent on the extent and location of nitration and the architecture of the resulting clot: some experimental studies suggest preserved fibrinolytic susceptibility, whereas others demonstrate delayed clot dissolution. Importantly, the consequences of fibrinogen nitration cannot be categorized as uniformly procoagulant or anticoagulant. Different degrees of modification may generate clots that form prematurely, are mechanically abnormal, or respond unpredictably to fibrinolytic stimuli [35]. The interactions of nitrated fibrinogen with platelets and endothelial cells also remain incompletely resolved, with experimental studies yielding partially divergent findings. Nevertheless, the presence of nitrated fibrinogen in conditions characterized by vascular inflammation, smoking, endotoxemia, sepsis, and cardiovascular disease supports its potential role as a mediator and a biomarker of thrombotic risk [36]. This review also considers therapeutic strategies aimed at restricting fibrinogen nitration. Natural polyphenols may limit nitrative damage by scavenging reactive intermediates and reducing tyrosyl radical formation, although their low systemic bioavailability remains a major obstacle. A more targeted approach involves inhibiting NADPH oxidases, thereby decreasing superoxide generation upstream and limiting peroxynitrite formation at its source. These observations reinforce a major theme of this Special Issue: successful redox therapy will probably require the precise targeting of oxidant-generating pathways rather than the indiscriminate administration of general antioxidants.

The authors of Contribution 2 examined the antiatherogenic actions of resveratrol using complementary in vitro, in vivo, and transcriptomic approaches. Atherosclerosis is sustained by the interaction among modified lipoproteins, endothelial activation, monocyte recruitment, macrophage differentiation, inflammasome activation, extracellular matrix degradation, and vascular smooth muscle cell remodeling [37,38,39]. ROS participate at almost every stage of this process, from LDL oxidation and inflammatory signaling to mitochondrial dysfunction and plaque destabilization [40]. The authors investigated resveratrol in human monocytes, macrophages, endothelial cells, and vascular smooth muscle cells as well as in LDL-receptor-deficient mice fed a high-fat diet. In vitro, resveratrol inhibited chemokine-driven monocyte migration, ROS production, inflammatory gene expression, inflammasome-dependent interleukin-1β release, matrix metalloproteinase activity, and pathological smooth muscle cell invasion [41,42]. These actions demonstrate that the biological effect of resveratrol extends well beyond direct chemical antioxidant activity. Resveratrol instead appears to influence interconnected inflammatory, metabolic, and redox-sensitive pathways in several cellular components of the atherosclerotic lesion.

In vivo, resveratrol reduced circulating LDL/VLDL cholesterol and cholesteryl ester levels and produced favorable changes in lymphoid cell populations. Its most prominent vascular effect, however, was not a reduction in plaque lipid content but a profound change in plaque composition. Resveratrol reduced macrophage and T-cell accumulation while increasing smooth muscle cell and collagen content, resulting in a more stable plaque phenotype. This distinction is clinically important. Treatment may reduce cardiovascular risk without producing a substantial decrease in plaque volume if the treatment transforms an inflamed, rupture-prone lesion into a more fibrotic and mechanically stable structure. The RNA sequencing of the thoracic aorta identified changes in pathways linked to oxidative phosphorylation, sirtuin signaling, cholesterol metabolism, NF-κB activation, T-cell responses, necroptosis, collagen synthesis, and plaque stability. These molecular data provide a mechanistic bridge between the cellular experiment results and the histological phenotype observed in vivo. The study also highlights the need to evaluate resveratrol in models of established-plaque regression and ultimately in adequately powered clinical trials. The molecular pathways identified through transcriptomic analyses will also require functional validation using targeted genetic or pharmacological approaches.

Whereas Contribution 2 addresses the modification of atherosclerotic biology, Contribution 3 covers the clinical monitoring of biological recovery in heart failure. Heart failure with improved ejection fraction (HFimpEF) is an increasingly recognized phenotype describing patients whose previously reduced left ventricular ejection fraction improves following treatment. However, an improvement in ejection fraction does not necessarily indicate complete myocardial recovery [43]. Persistent neurohormonal activation, inflammation, oxidative stress, endothelial dysfunction, fibrosis, or thrombotic activation may remain even when conventional imaging parameters improve. In this real-life study, 100 outpatients with HFimpEF underwent clinical and laboratory evaluation at baseline and after six months of pharmacological optimization. Treatment included, where clinically appropriate, the optimization of angiotensin-receptor–neprilysin inhibition, mineralocorticoid receptor antagonists, and sodium–glucose cotransporter-2 inhibitors. The investigators assessed biomarkers representing oxidative stress, inflammation, platelet activation, neurohormonal signaling, and metabolic and renal function. Proteomic analysis was additionally performed in a subgroup of 13 patients.

After six months, improvements were observed in glycometabolic, renal, inflammatory, and cardiovascular biomarker profiles. The proteomic analysis identified selective changes in proteins associated with cardiovascular remodeling and endothelial biology, including insulin-like growth factor-binding protein 4, thrombospondin-4, intercellular adhesion molecule 1, and syndecan-4. These findings suggest that contemporary heart failure therapy exerts effects extending beyond hemodynamic unloading and ejection-fraction recovery, influencing oxidative stress, inflammation, vascular activation, extracellular matrix biology, and thrombotic risk.

The study also demonstrates why a multimarker approach may be more informative than reliance on natriuretic peptides or ejection fraction alone. Biomarkers such as NOX2 and 8-isoprostane may help identify patients in whom structural improvement is not accompanied by complete redox recovery [44]. Such patients might remain at risk of clinical deterioration despite an apparently favorable echocardiographic response. Serial biomarker evaluation could therefore contribute to treatment titration, risk stratification, and the identification of incomplete biological remission.

Contribution 4 considers a different heart failure phenotype: heart failure with preserved ejection fraction (HFpEF). HFpEF is characterized by marked clinical and biological heterogeneity and frequently coexists with aging, obesity, hypertension, diabetes, renal dysfunction, and atrial fibrillation. These comorbidities sustain systemic inflammation, coronary microvascular dysfunction, impaired nitric oxide signaling, mitochondrial dysfunction, myocardial stiffness, and interstitial fibrosis. The resulting syndrome is therefore not simply a disorder of diastolic mechanics but a systemic cardiometabolic disease [45]. The authors review the potential of dietary polyphenols as adjunctive agents in HFpEF, with particular attention to phenolic acids, flavonoids, stilbenes, and lignans. Although structurally diverse, these compounds converge on several pathways relevant to HFpEF. The activation of the Keap1/Nrf2 axis increases the expression of endogenous antioxidant enzymes, including superoxide dismutase, catalase, glutathione peroxidase, heme oxygenase-1, and NAD(P)H quinone dehydrogenase 1. In parallel, the suppression of NF-κB-mediated signaling may reduce cytokine production and chronic vascular inflammation.

Polyphenols may also activate AMPK and SIRT1, supporting mitochondrial biogenesis, metabolic flexibility, autophagy, and cellular stress resistance [46,47,48]. Their effects on PI3K/Akt/eNOS signaling may restore nitric oxide availability and endothelial function, whereas the inhibition of TGF-β-dependent pathways could attenuate collagen deposition and myocardial fibrosis. The modulation of calcium handling, titin stiffness, lipid oxidation, apoptosis, and extracellular matrix turnover further supports the biological plausibility of polyphenols as multidimensional interventions in HFpEF.

An additional element in heart failure is the interaction between polyphenols and the gut microbiota. Microbial metabolism can generate bioactive phenolic derivatives and short-chain fatty acids that influence inflammation, mitochondrial function, and cardiometabolic homeostasis. This interaction may be particularly relevant in obesity-related HFpEF, in which intestinal dysbiosis, systemic inflammation, and metabolic dysfunction are closely linked [49]. However, the strength of the mechanistic rationale contrasts the weakness of the available clinical evidence. Most data have been derived from experimental models, observational nutritional studies, or populations with reduced-ejection-fraction heart failure or general metabolic disease. Randomized studies specifically enrolling well-phenotyped patients with HFpEF are scarce. Low absorption, rapid metabolism, heterogeneous formulations, inconsistent dosing, and the biological diversity of HFpEF further complicate translation. Polyphenols should consequently be viewed as potential adjunctive modulators rather than substitutes for guideline-directed treatment. Future trials should stratify participants according to cardiometabolic phenotype and incorporate biomarkers of oxidative stress, endothelial function, inflammation, fibrosis, and mitochondrial activity alongside conventional clinical outcomes.

Contribution 5 focuses on atherosclerosis, highlighting the essential pharmacological concept that the molecule consumed in food may not be the molecule that ultimately reaches the cardiovascular system [50]. Punicalagin, an abundant ellagitannin in pomegranate, is poorly absorbed and undergoes conversion to ellagic acid and subsequently to urolithins through the action of the intestinal microbiota. The cardiovascular effects associated with pomegranate intake may therefore substantially depend on microbial metabolites rather than exclusively on the parent polyphenol. The authors compared punicalagin, ellagic acid, and urolithins A, B, C, and D in several cellular processes implicated in atherogenesis. These compounds showed distinct rather than interchangeable, biological profiles. Punicalagin and selected metabolites inhibited ROS production, monocyte migration, modified-LDL uptake, macropinocytosis, and inflammatory gene expression in macrophages and endothelial cells. Some responses were compound-specific, and not every metabolite was uniformly protective. This observation cautions against treating “polyphenols” as a homogeneous pharmacological class.

Urolithin A displayed the most consistent antiatherogenic activity and was therefore selected for in vivo evaluation in high-fat-fed LDL-receptor-deficient mice. In this model, urolithin A reduced plaque content, lesion size, and vascular occlusion without notably changing the plaque lipid content. Urolithin A also decreased macrophage and T-cell accumulation while increasing smooth muscle cells and collagen, resulting in a higher plaque stability index. As in the resveratrol study, protection was therefore associated not only with lesion burden but also with the suppression of vascular inflammation and the favorable remodeling of plaque composition. The transcriptome therefore connects the metabolism of dietary polyphenols by the gut microbiota with systemic immune regulation and structural changes in the arterial wall. This also raises the possibility that interindividual differences in gut microbial composition (the metabotype) determine who produces urolithin A and who is most likely to benefit from ellagitannin-rich foods.

These findings support the direct evaluation of urolithin A as a nutraceutical or pharmacological candidate rather than assuming that supplementation with poorly absorbed parent compounds generates equivalent exposure in every individual. Human trials will need to account for microbiome composition, metabolite generation, dose, formulation, and circulating concentrations. The work also illustrates how nutritional cardiology is moving toward a precision framework in which host metabolism and microbial biotransformation are considered integral components of therapeutic efficacy.

The final contribution expands the scope of antioxidant cardiovascular medicine from the treatment of established disease to prevention across the life course (Contribution 6). This review positions resveratrol within the integrated framework of cardiovascular–kidney–metabolic syndrome and the developmental origins of health and disease. These concepts recognize that cardiovascular disease is the cumulative outcome of interactions among metabolic, renal, and vascular dysfunction that may begin during prenatal or early postnatal development [51]. Within this framework, oxidative stress is not simply a late consequence of cardiovascular disease but a mechanism capable of altering developmental trajectories. Adverse early-life exposures can influence nephron number, vascular structure, mitochondrial function, insulin sensitivity, blood pressure regulation, and inflammatory responses. These changes may remain clinically silent for many years before contributing to hypertension, chronic kidney disease, obesity, diabetes, or overt cardiovascular disease.

The review emphasizes that resveratrol predominantly functions as a redox-responsive regulator rather than as a conventional free-radical scavenger in vivo. Resveratrol activates Nrf2–antioxidant response element signaling; modulates SIRT1, AMPK, PGC-1α, Akt, NF-κB, and mTOR; improves mitochondrial quality control; and restores nitric oxide bioavailability. Through these interconnected pathways, resveratrol may influence endothelial function, metabolic flexibility, inflammation, fibrosis, vascular stiffness, and pathological cardiac remodeling.

Preclinical studies suggest that resveratrol administration during gestation or lactation reprograms the pathways associated with later hypertension, renal dysfunction, obesity, and insulin resistance. However, these findings should not be interpreted as sufficient evidence for maternal supplementation in humans. Developmental timing, dose, placental transfer, long-term safety, sex-specific responses, and potential effects on normal redox-dependent development must be carefully evaluated before clinical translation. The major pharmacokinetic limitation remains the rapid metabolism and low circulating concentration of unconjugated resveratrol [52]. The review discusses several approaches designed to overcome this barrier, including chemical derivatives, nanoencapsulation, liposomes, polymeric micelles, and resveratrol–short-chain fatty acid ester hybrids. These strategies may increase stability, tissue delivery, and sustained release, but they also generate new questions regarding dose equivalence, metabolite activity, and long-term safety.

Artificial intelligence and systems biology approaches may assist in integrating gene–disease networks, identifying candidate targets, optimizing delivery systems, and stratifying patients. Nevertheless, the authors appropriately stress that computational predictions are tools for hypothesis generation and cannot replace experimental validation or carefully designed clinical trials. The future value of artificial intelligence in this field will depend on the quality of the biological data, the reproducibility of predicted mechanisms, and the use of clinically meaningful endpoints.

Taken together, the contributions to this Special Issue reveal several emerging directions in antioxidant cardiovascular medicine. First, oxidative and nitrosative modifications should be regarded as active disease mechanisms and potential biomarkers rather than nonspecific signs of tissue injury. Fibrinogen nitration exemplifies how a defined redox modification may directly influence thrombotic phenotype. 

Second, the term “antioxidant” should increasingly be used to describe the modulation of redox networks rather than the simple chemical neutralization of free radicals. Resveratrol, urolithin A, and other polyphenols exert their effects through transcriptional regulation, mitochondrial adaptation, inflammatory signaling suppression, lipid metabolism regulation, and nitric oxide pathway preservation. Their benefits cannot be adequately predicted from in vitro radical scavenging capacity alone.

Third, treatment success should not be exclusively evaluated through conventional structural endpoints. Plaque composition may be more relevant than plaque size in some settings, and recovery from heart failure may require more than an increase in ejection fraction. Histological measures of plaque stability and longitudinal molecular biomarker profiles can reveal therapeutic effects overlooked by conventional imaging or laboratory parameters.

Fourth, bioavailability and biotransformation are central determinants of treatment efficacy. The therapeutic effect of a dietary compound depends on absorption, metabolism, the tissue distribution, the formulation, and the gut microbiota. Parent polyphenols and their metabolites may have different, and occasionally opposing, biological properties. Future trials must therefore measure exposure to active metabolites and account for individual metabolic and microbial variability.

Finally, the evidence supports a transition toward biomarker-guided, phenotype-specific, and potentially life-course interventions. The indiscriminate administration of antioxidant supplements is unlikely to produce consistent cardiovascular benefit. More promising strategies will identify patients with defined redox abnormalities, select compounds targeting the relevant pathway, establish biologically effective exposure, and monitor the restoration of redox balance over time.

The studies gathered in this Special Issue demonstrate that redox biology intersects with every stage of cardiovascular disease, from early developmental programming to endothelial dysfunction, atherosclerotic plaque formation, thrombosis, myocardial remodeling, and heart failure. Translating these insights into clinical benefit will require rigorous pharmacokinetic studies, standardized biomarkers, mechanistically informed clinical trials, and close collaboration among redox biologists, cardiologists, pharmacologists, nutrition scientists, and computational researchers.

Antioxidant cardiovascular medicine is therefore entering a new phase. Its future will not be defined by the search for a universal antioxidant but by the capacity to understand when, where, and how redox signaling becomes pathological and to intervene with sufficient molecular and clinical precision to restore balance without disrupting its essential physiological functions.

## Data Availability

No new data were created or analyzed in this study. Data sharing is not applicable to this article.

## Abbreviations

The following abbreviations are used in this manuscript:

ROS	Reactive oxygen species
RNS	Reactive nitrogen species
NADPH	Nicotinamide adenine dinucleotide phosphate
LDL	Low-density lipoproteins
VLDL	Very-low-density lipoproteins
HFpEF	Heart failure with preserved ejection fraction
Keap1	Kelch-like ECH-associated protein 1
Nrf2	Nuclear factor erythroid 2-related factor 2
NF-kB	Nuclear factor kappa-light-chain-enhancer of activated B cells
AMPK	AMP-activated protein kinase
SIRT1	Silent mating type information regulation 2 homolog 1
PI3K	Phosphoinositide 3-kinase
eNOS	Endothelial nitric oxide synthase
PGC-1α	Peroxisome proliferator-activated receptor gamma coactivator 1-alpha
TGF-β	Transforming growth factor-beta
mTOR	Mammalian target of rapamycin
